# The impact of Medicare copayments for skilled nursing facilities on length of stay, outcomes, and costs

**DOI:** 10.1111/1475-6773.13227

**Published:** 2019-10-27

**Authors:** Rachel M. Werner, R. Tamara Konetzka, Mingyu Qi, Norma B. Coe

**Affiliations:** ^1^ Department of Medicine Perelman School of Medicine University of Pennsylvania Philadelphia Pennsylvania; ^2^ Leonard Davis Institute of Health Economics University of Pennsylvania Philadelphia Pennsylvania; ^3^ Center for Health Equity Research and Promotion Crescenz VA Medical Center Philadelphia Pennsylvania; ^4^ Department of Public Health Sciences University of Chicago Chicago Illinois; ^5^ Department of Medical Ethics and Health Policy Perelman School of Medicine University of Pennsylvania Philadelphia Pennsylvania

**Keywords:** Medicare payment, postacute care, skilled nursing facility

## Abstract

**Objective:**

To investigate the impact of Medicare's skilled nursing facility (SNF) copayment policy, with a large increase in the daily copayment rate on the 20th day of a benefit period, on length of stay, patient outcomes, and costs.

**Data Sources and Study Setting:**

Retrospective cohort study from 2012 to 2016 using Medicare claims and SNF assessment data, including SNF admissions for Medicare fee‐for‐service beneficiaries.

**Study Design:**

We first estimate how changes in Medicare's SNF copayment on the 21st day of a patient's benefit period affect length of SNF stay. We then use benefit day on admission as an instrumental variable to estimate the impact of SNF length of stay related to the copayment policy on readmission and Medicare payment.

**Principal Findings:**

From 2012 to 2016, we examined 291 134 SNF admissions. Higher benefit day on SNF admission was strongly associated with shorter SNF stays. A 1‐day shorter SNF stay was associated with higher readmission rate within 30 days of hospital discharge (1.5 percentage points; 95% CI 1.4‐1.6, *P *< .001) and within 30 days of SNF discharge (0.9 percentage points; 95% CI 0.8‐1.0), lower total Medicare payment for the 90‐day episode after hospital discharge ($396; 95% CI 361‐431, *P *< .001), but $179 higher payment for the 90 days after SNF discharge (95% CI 149‐210, *P *< .001), offsetting the lower payment for the shorter index SNF stay.

**Conclusions:**

Medicare's SNF copayment policy is associated with shorter lengths of stay and worse patient outcomes, suggesting the copayment policy has unintended and negative effects on patient outcomes.

## 1 INTRODUCTION

Use of skilled nursing facilities (SNFs) for postacute care is common and costly. Institutional postacute care incurs enormous costs to Medicare^1^ and is a large contributor to geographic variation in Medicare spending.^2^ In 2015, Medicare spent over $60 billion on postacute care,^3^ an amount that has rapidly increased in recent years. Over 40 percent of Medicare beneficiaries receive postacute care after a hospital discharge,^1^ the majority of which is in a SNF.^3^, ^4^

In an effort to reduce unnecessary SNF utilization, Medicare has a long‐standing copayment policy for fee‐for‐service beneficiaries where Medicare pays in full for the first 20 days of a SNF benefit period, but on the 21st day, daily copayments increase from $0 to over $150.^5^ Our prior work demonstrated a large increase in the number of patients discharged on their 20th day of a SNF stay.^6^ Because patients do not face financial pressures to shorten their stay until their 21st day, it is possible that SNF stays are longer than necessary, particularly because Medicare payment to SNFs is per diem, giving SNFs little incentive to reduce length of stay. These factors may lead to unnecessary additional SNF days and wasteful spending if there is no benefit to patients. On the other hand, with the large increase in copayment on the 21st day of a SNF stay, the associated financial pressures may result in lengths of stay that are shorter than optimal. Recent research has shown that sending patients home with home health care results in worse outcomes for patients than discharging them to SNF,^7^ which may imply that insufficient length of SNF stay would also result in worse outcomes. In this case, cost savings from shortening SNF stays would have to be balanced against adverse consequences for patients to determine overall welfare effects. In addition, any savings from shorter SNF stays may be offset by higher spending after SNF discharge.

Clarifying the impact of Medicare's copayment policy on SNF length of stay and on patient outcomes will help inform payment policy around SNF care and health care policies aimed at optimizing patient outcomes while constraining costs. This is particularly important in the setting of recent payment reforms that target postacute care and have been associated with both declines in SNF utilization and shorter SNF stays.^8^, ^9^, ^10^, ^11^ Our objective was therefore to investigate the relationship between Medicare's SNF copayment policy and SNF length of stay and also between patient outcomes and Medicare costs.

## 2 METHODS

### 2.1 Data

We used Medicare data to observe all Medicare‐reimbursed fee‐for‐service skilled nursing facility admissions in the United States between 2012 and 2016. These data were supplemented with the Medicare Beneficiary Summary File containing information on beneficiary enrollment in Medicare, the Minimum Data Set containing assessment data for all SNF stays to measure patient characteristics on SNF admission, and the American Community Survey containing ZIP‐code‐level socioeconomic characteristics for each Medicare beneficiary's ZIP code of residence.

### 2.2 Study variables

We started with all Medicare fee‐for‐service beneficiaries admitted to a SNF within 24 hours of a hospital discharge between January 1, 2012, and October 1, 2016 (n = 7 110 815). We excluded discharges for beneficiaries who received hospice (n = 255 536), who had an MDS‐reported life expectancy of <6 months (n = 68 814), whose hospitalization was preceded by a long‐term care stay in a nursing home in the prior 100 days (n = 236 703), who were ineligible for the CMS‐defined unplanned readmission measure, which is our main outcome of interest (n = 947 645), or who had missing values for zip code, SNF resource utilization group (RUG), or benefit day (n = 15 931), leaving 5 586 186 SNF admissions.

Using this cohort of SNF admissions, we measured SNF length of stay for each admission, measured in the number of days that Medicare paid for a SNF stay, and also defined each patient's SNF benefit period. Fee‐for‐service Medicare covers a maximum of 100 days per SNF benefit period. It fully pays for SNF care for the first 20 days within a benefit period, but on days 21‐100, most patients are responsible for a daily copayment of over $150.^5^ The benefit period begins on the day of admission to a SNF, lasts up to 100 days, can span multiple SNF stays, and ends when there has been no inpatient hospital care or additional SNF care for 60 consecutive days. For each SNF admission, we measured the day of the benefit period at SNF admission, taking into account prior hospital and SNF stays. We use this complete cohort to describe the relationship between SNF length of stay and the copayment increase on the 21st day of a SNF benefit period.

Our patient outcome of interest was readmission. We first defined readmission within 30 and 90 days of hospital discharge by including all patients discharged alive from the hospital and admitted to SNF within 24 hours of hospital discharge. We next defined readmissions within 30 and 90 days of SNF discharge to ensure that our exposure of interest, SNF length of stay, was not being partially determined by the outcome of readmission. For this measure, we included all patients who were discharged alive from the SNF and were not readmitted to the hospital on the same day of SNF discharge (ie, those patients who went directly from hospital to SNF). In all cases, we based our measure on Medicare's definition of hospital‐wide readmission from the Hospital Readmission Reduction Program,^12^ including unplanned readmissions to any acute care hospital within 30 or 90 days of hospital or SNF discharge. We also examined successful discharge from SNF, a composite outcome defined as patients who were discharged from SNF and who did not die or have additional hospital or SNF claim within 30 days of SNF discharge.

We also examined Medicare payments as an outcome. These payments were measured as (a) total Medicare Part A payments over the hospitalization and 90 days after discharge, including all payments for postacute care and rehospitalizations, (b) payment for the index hospitalization (which we expect to be unaffected by SNF length of stay given that payment is determined prospectively based on the diagnosis‐related group [DRG]), (c) payment for the initial SNF stay after hospital discharge, and (d) total Part A payments over the first 90 days after SNF discharge.

We included patient‐level covariates in all regressions, including age, sex, race, marital status, total Medicare Part A payments in the year prior to hospitalization, and 31 indicators of comorbidities based on CMS Hospital Readmission Reduction Program specifications. We also included ZIP‐code‐level measures of socioeconomic status (median household income, percentage living in poverty, and unemployment rate), information on the index hospitalization (length of stay in the hospital and DRG fixed effects), fixed effects of the resource utilization group (RUG) of the SNF stay, and year and SNF fixed effects to account for unobserved time‐invariant heterogeneity across SNFs that may be related to both the SNF length of stay and outcomes of interest. In regressions of readmission and total payment, we accounted for censoring by patient death by adjusting for the number of days each person is alive in that period over which the outcome is measured.

### 2.3 Statistical analyses

We first describe the relationship between a patient's SNF benefit day on discharge from SNF and length of stay in a SNF.

The estimated relationship between SNF length of stay and outcomes may be biased because of unobserved patient illness severity. For example, patients who stay longer in a SNF may also have worse outcomes because they are sicker in ways we cannot observe in the data, which could lead to an underestimate of the benefits of a longer stay. Alternatively, patients who stay longer in a SNF may be those with more resources who are likely to have lower illness severity in ways we cannot observe in the data, which could lead to an overestimate of the benefits of a longer stay.

A patient's benefit day is a strong predictor of length of SNF stay because when copays increase, patients are more likely to be discharged home.^6^ Therefore, for each SNF admission, we used the day of the benefit period at SNF admission as an instrumental variable to predict SNF length of stay. The idea behind this approach is to identify a portion of the variation in SNF length of stay that is driven by something that is unrelated to patient health (or other endogenous factors) and is therefore not subject to bias from unobservable patient characteristics, and then to relate this exogenous variation to variations in patient outcomes. By using benefit day on SNF admission as our instrument, we leveraged the change in Medicare copayments on the 21st day of a patient's benefit period as an instrumental variable to isolate exogenous variation in SNF length of stay. In addition, because our identification came from the change in copayments that are part of Medicare's SNF copayment policy, it enabled us to estimate the differences in outcomes that are specifically associated with variation in lengths of stay from the payment policy, giving us an unbiased estimate of the effect of payment policy‐associated length of stay on patient outcomes.

For patients admitted to a SNF at the beginning of a new benefit period, their benefit day on SNF discharge equals their SNF length of stay, providing no exogenous variation in length of stay. Therefore, to estimate the relationship between SNF length of stay and outcomes, we defined a smaller study cohort that allowed us to leverage variation in the SNF benefit day on SNF admission as an instrumental variable, and isolate exogenous variation in SNF length of stay related to the copayment policy.

We did this by focusing on the approximately 20 percent of SNF admissions for Medicare beneficiaries who already had used one or more SNF benefit days at the time of SNF admission (excluding 4 487 277 SNF stays). We further focused on SNF admissions for beneficiaries discharged on benefit days 1‐40 (excluding 701 581 discharges), as patients who stay in a SNF for more than 40 days are likely more severely ill than the typical SNF patients and have also stayed well beyond benefit day 21, when the copayment increases, which is used to help identify the effect of interest. Thus, the length of stay for the excluded discharges is unlikely to be affected by the day 21 copayment change. Finally, we excluded discharges for beneficiaries who were dual‐eligible and therefore not responsible for the SNF copayment (n = 106 194). Our final study cohort was 291 134 SNF admissions. This cohort allowed us to focus on the effect of Medicare's copayment policy and also improves the identification of the effect of SNF length of stay on patient outcomes and Medicare cost, though decreases generalizability.

In this cohort, we examined the relationship between SNF benefit day on admission and SNF length of stay using multivariable linear regression and adjusting for the covariates listed above and standard errors for clustering within SNF. We found that SNF benefit day on admission was a very strong predictor of SNF length of stay—a 1‐day increase in benefit day on admission was strongly associated with a SNF length of stay that was 0.3 days shorter (*F*‐statistic 30 182; Table 2),^13^ providing validity for this instrument in terms of its predictive power.

We next examined the relationship between the instrument and measured confounders, as balance in confounders across values of the instrument lends credibility to the instrument (Table S1). We found that most patient covariates were relatively well balanced, with the most remaining imbalances in measures of clinical severity (total number of comorbidities and Medicare spending in the prior year). We accounted for residual imbalances by adjusting for them in the instrumental variable model, as the instrumental variable is valid if it is uncorrelated with unobserved confounders, conditional on observable confounders. We also tested the sensitivity of our results to this imbalance, described below.

We used an instrumental variable approach to estimate the effect of differences in SNF length of stay around the copayment bump on patient outcomes. This was implemented using 2‐stage least‐squares regressions (2SLS) where the first stage predicted the effect of SNF benefit day on admission (the instrument) on SNF length of stay and the second stage estimated the relationship between predicted length of stay from the first stage and the outcomes of interest. Both stages adjusted for covariates described above and standard errors for clustering within SNF. The effects were estimated for the study cohort of patients described above. We also estimated the impact of SNF length of stay on each outcome in subsets of patients hospitalized with the five most common DRGs being discharged to SNF: total knee or hip replacement, sepsis, urinary tract infection, hip fracture, and congestive heart failure.

We conducted a number of sensitivity analyses. To further address the impact of imbalances in illness severity, we did two additional analyses. First, we stratified this sample by illness severity, a common approach to testing the sensitivity of results to an imbalanced covariate.^14^ We stratified the cohort using the median number of comorbidities—six comorbidities—and re‐estimated the IV model within these strata. Patient covariates were better balanced within these strata (Table S2), and the *F*‐statistic for the instrument was 11 631.6 and 19 398.9 in the high‐ and low‐clinical‐severity cohorts, respectively. Second, we re‐estimated the IV model in the subgroup of SNF admissions that were more than 30 days after the beneficiary's prior SNF discharge and thus required a new hospital stay prior to SNF admission (but <60 days so it was still in the same benefit period). Thus, these second SNF stays were more plausibly a new episode of care within the same benefit period. The balance of patient covariates for this subgroup was also better (Table S3), and the *F*‐statistic for the instrument was 1621.7.

We finally re‐estimated the IV model in a cohort that included a narrower range of benefit days on SNF discharge, benefit days 16‐25. While this reduces generalizability, it is focused closer to the discontinuity in copayment that occurs after the 20th benefit day. The *F*‐statistic for the instrument in this sample was 172 540.5. Covariate balance for this sample is included in Table S4.

## 3 RESULTS

From 2012 to 2016, our study cohort included 291 134 admissions to SNF for Medicare beneficiaries. Most discharges were female (57.8 percent) and white (90.5 percent), and 40.3 percent were married (Table 1). Compared to the larger SNF cohort from which we drew this sample, the study cohort of patients admitted in the middle of a SNF benefit period had a lower percentage of female and black patients and were sicker on average.

**Table 1:** Patient characteristics of the study cohort (those admitted to a SNF in the middle of a benefit period and discharged from SNF on benefit day 1‐40 d) and of the larger cohort of all SNF admissions from which the study cohort was drawn

	Study cohort n = 291 134	All SNF admissions n = 5 586 186
Age, mean (SD)	81.7 (8.1)	81.8 (8.2)
Female, n (%)	168 368 (57.8%)	3 632 861 (65.0%)
Race, n (%)		
White	263 434 (90.5%)	4 858 412 (87.0%)
Black	21 168 (7.3%)	501 323 (9.0%)
Hispanic	1451 (0.5%)	76 274 (1.4%)
Married, n (%)	117 239 (40.3%)	1 871 689 (33.5%)
Median household income of ZIP code, mean (SD)	61 453 (24 597)	59 336 (24 104)
Percent of unemployment of ZIP code, mean (SD)	7.2 (3.5)	7.4 (3.6)
Percent of population living in poverty of ZIP code, mean (SD)	13.3 (8.2)	14.3 (8.7)
No. of comorbidities, mean (SD)	5.9 (2.8)	3.6 (2.9)
Total Medicare Part A spending in prior year, mean (SD)	41 992 (35 713)	26 636 (31 038)
Hospital length of stay, mean (SD)	6.1 (5.0)	6.3 (5.2)
Five most common diagnosis‐related groups, n (%)		
Total knee or hip replacement	20 667 (7.1%)	566 598 (10.1%)
Sepsis	20 428 (7.0%)	312 237 (5.6%)
Hip fracture	6527 (2.5%)	154 358 (2.8%)
Urinary tract infection	7002 (2.4%)	152 242 (2.7%)
Congestive heart failure	6527 (2.2%)	123 907 (2.2%)

Abbreviations: SD, standard deviation; SNF, skilled nursing facility.

**Figure 1:**
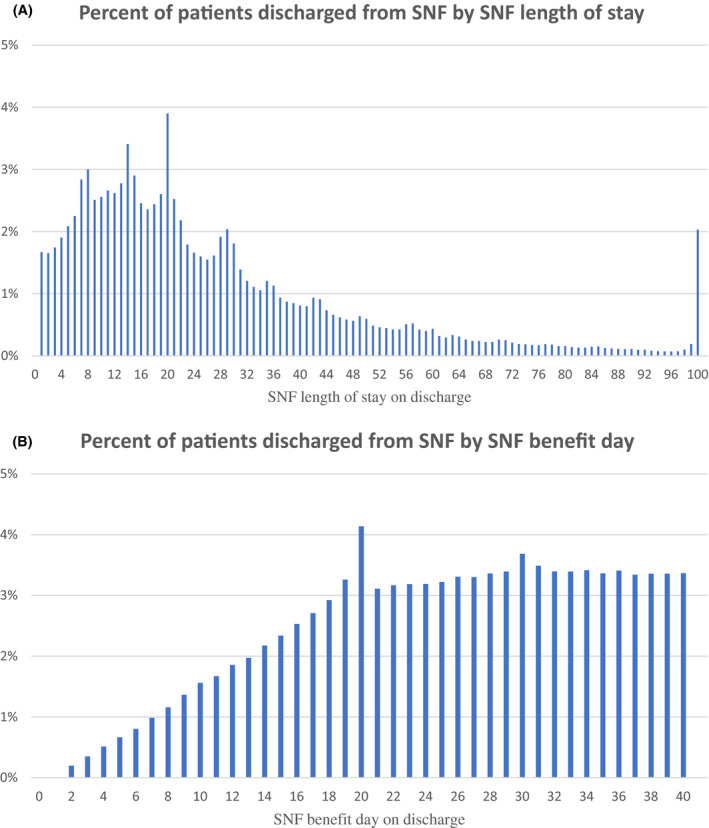
A, Histogram of percentage of patients discharged from SNF by SNF length of stay among all SNF discharges (n = 5 586 186). B, Histogram of the percentage of patients discharged from SNF on each SNF benefit day, limiting the cohort to those beneficiaries who have already used 1 or more benefit days in their benefit period at the time of SNF admission (the study cohort, n = 291 134). SNF, skilled nursing facility [Color figure can be viewed at http://www.wileyonlinelibrary.com/]

We first examined the relationship between SNF length of stay and the increase in copayment on day 21 of the benefit period. Among the full cohort of all patients admitted to a SNF, the most common day for SNF discharge was on the 20th day, with 3.7 percent of all admissions being discharged on that day. By day 20, 43.3 percent of all SNF admissions had been discharged (Figure 1A). After limiting the sample to our study cohort—those beneficiaries admitted in the middle of a benefit period and focusing on those discharged on benefit day 1‐40—we observed a similar pattern. The most common benefit day for SNF discharge was still the 20th day, with 4.1 percent of this cohort being discharged on that day. By day 20, 33.2 percent of all SNF admissions in this cohort had been discharged (Figure 1B).

In multivariable regression, there was a strong relationship between benefit day on SNF admission and SNF length of stay, where the further along someone was in their SNF benefit period at SNF admission, the shorter their SNF length of stay was. A 1‐day increase in benefit day on admission was strongly associated with a SNF length of stay that was 0.3 days shorter (95% CI 0.31‐0.31, *P* < .001; Table 2).

**Table 2:** Relationship between benefit day on SNF admission and SNF length of stay in study cohort (1st stage of 2‐stage least‐squares analysis)

	Difference (95% confidence intervals)	*P*‐value	Partial *F*‐statistic
SNF length of stay with one additional benefit day on SNF admission, days	−0.31 (−0.31 to −0.31)	<.001	30 182

Abbreviation: SNF, skilled nursing facility.

Outcomes for the study cohort are summarized in Table 3. In the study cohort, the readmission rate within 30 and 90 days of hospital discharge was 29.3 and 39.1 percent, respectively, and the readmission rate within 30 and 90 days of SNF discharge was 36.7 and 43.1 percent, respectively. Close to half of patients who were discharged from SNF remained out of the SNF or hospital and alive for 30 days. The mean total Medicare payment was $26 405 for the 90‐day episode after hospital discharge and $11 933 over the 90 days after SNF discharge. Compared to all patients admitted to a SNF, our study cohort had substantially worse patient outcomes.

**Table 3:** Unadjusted patient outcomes and Medicare payment of the study cohort (those admitted to a SNF in the middle of a benefit period discharged from SNF on benefit day 1 to 40 d) and of the larger cohort of all SNF admissions from which the study cohort was drawn

Patient outcomes	Study cohort n = 291 134	All SNF admissions n = 5 586 186
	%	%
Readmission within 30 d of hospital discharge, percentage points	29.3	13.9
Readmission within 90 d of hospital discharge, percentage points	39.1	25.4
Readmission within 30 d of SNF discharge, percentage points	36.7	21.6
Readmission within 90 d of SNF discharge, percentage points	43.1	26.4
Successful discharge from SNF	48.9	68.3

Medicare payment	Mean (SD)	Mean (SD)
Total Medicare payment in first 90 d after hospital discharge, $	26 405 (22 556)	28 364 (21 137)
Medicare payment for index hospitalization, $	9956 (22 556)	10 930 (11 633)
Medicare payment for index SNF stay, $	5253 (4247)	10 683 (9194)
Total Medicare payment within first 90 d after SNF discharge, $	11 933 (19 720)	8044 (16 267)

Abbreviations: SD, standard deviation; SNF, skilled nursing facility.

We then examined the relationship between SNF length of stay and patient outcomes and Medicare costs. We first estimated multivariable regression using ordinary least squares, finding that shorter lengths of stay were associated with worse outcomes (or, conversely, that longer lengths of stay were associated with better outcomes). One day longer in a SNF was associated with lower readmissions within 30 and 90 days of hospital discharge and also within 30 and 90 days of SNF discharge (Table 4, column 1). In multivariable regression, longer SNF stays were also associated with lower episode spending both in the 90 days following hospital discharge and in the 90 days after SNF discharge.

**Table 4:** Differences in outcome with one additional day in SNF using limited sample of patients (excluding dual‐eligible patients) discharged on benefit days 1‐40, from multivariable and instrumental variable regressions

	Multivariable regression: study cohort		IV regression: study cohort		IV regression: total knee or hip replacement		IV regression: sepsis		IV regression: urinary tract infection		IV regression: hip fracture		IV regression: congestive heart failure	
	Difference (95% CI)	*P*‐value	Difference (95% CI)	*P*‐value	Difference (95% CI)	*P*‐value	Difference (95% CI)	*P*‐value	Difference (95% CI)	*P*‐value	Difference (95% CI)	*P*‐value	Difference (95% CI)	*P*‐value
Readmission within 30 d of hospital discharge, percentage points	−1.9 (−1.9 to −1.8)	<.001	−1.5 (−1.6 to −1.4)	<.001	−2.1 (−3.1 to −1.1)	<.001	−1.5 (−1.8 to −1.2)	<.001	−1.4 (−2.2 to −0.7)	<.001	−2.2 (−3.7 to −0.7)	.004	−1.7 (−2.1 to −1.2)	<.001
	n = 291 134		n = 291 134		n = 5052		n = 20 428		n = 6527		n = 1766		n = 12 252	
Readmission within 90 d of hospital discharge, percentage points	−1.2 (−1.2 to −1.2)	<.001	−1.1 (−1.1 to −1.0)	<.001	−1.5 (−2.7 to −0.3)	.013	−0.8 (−1.1 to −0.4)	<.001	−1.0 (−1.8 to −0.3)	.009	−0.6 (−2.2 to 0.9)	.405	−1.0 (−1.5 to −0.5)	<.001
	n = 291 134		n = 291 134		n = 5052		n = 20 428		n = 6527		n = 1766		n = 12 252	
Readmission within 30 d of SNF discharge, percentage points	−1.2 (−1.2 to −1.1)	<.001	−0.9 (−1.0 to −0.8)	<.001	−1.9 (−3.0 to −0.8)	<.001	−0.9 (−1.3 to −0.5)	<.001	−1.0 (−1.8 to −0.2)	.010	−1.3 (−2.9 to 0.2)	.094	−1.0 (−1.6 to −0.4)	<.001
	n = 263 396		n = 263 396		n = 4959		n = 16 822		n = 6110		n = 1641		n = 10 429	
Readmission within 90 d of SNF discharge, percentage points	−1.1 (−1.2 to −1.1)	<.001	−1.1 (−1.1 to −1.0)	<.001	−1.6 (−2.8 to −0.4)	.007	−1.0 (−1.4 to −0.6)	<.001	−1.1 (−1.9 to −0.3)	.010	−0.9 (−2.5 to 0.7)	.266	−1.0 (−1.6 to −0.3)	.002
	n = 263 396		n = 263 396		n = 4959		n = 16 822		n = 6110		n = 1641		n = 10 429	
Successful discharge from SNF	1.4 (1.4 to 1.4)	<.001	1.4 (1.3 to 1.5)	<.001	2.8 (1.6 to 4.0)	<.001	1.4 (1.0 to 1.8)	<.001	1.2 (0.5 to 2.0)	.002	1.2 (0.0 to 2.5)	.053	1.4 (0.8 to 1.9)	<.001
	n = 263 396		n = 263 396		n = 4959		n = 16 822		n = 6110		n = 1641		n = 10 429	
Total Medicare payment in first 90 d after hospital discharge, $	−57 (−68 to −47)	<.001	396 (361 to 431)	<.001	818 (413 to 1223)	<.001	471 (323 to 619)	<.001	527 (271 to 783)	<.001	171 (−358 to 701)	.527	309 (81 to 538)	.008
	n = 291 134		n = 291 134		n = 5052		n = 20 428		n = 6527		n = 1766		n = 12 252	
Medicare payment for index hospitalization, $	16 (13 to 19)	<.001	97 (78 to 115)	<.001	275 (162 to 388)	<.001	77 (41 to 113)	<.001	17 (−21 to 56)	.375	108 (1 to 216)	.048	87 (45 to 129)	<.001
	n = 291 134		n = 291 134		n = 5052		n = 20 428		n = 6527		n = 1766		n = 12 252	
Medicare payment for index SNF stay, $	392 (390 to 395)	<.001	591 (585 to 596)	<.001	912 (820 to 1004)	<.001	594 (570 to 618)	<.001	597 (548 to 647)	<.001	555 (442 to 668)	<.001	588 (554 to 622)	<.001
	n = 291 134		n = 291 134		n = 5052		n = 20 428		n = 6527		n = 1766		n = 12 252	
Total Medicare payment within first 90 d after SNF discharge, $	−364 (−374 to −354)	<.001	−179 (−210 to −149)	<.001	−287 (−695 to 121)	.168	−52 (−195 to 90)	.473	−15 (−277 to 248)	.912	−334 (−866 to 198)	.219	−252 (−486 to −18)	.035
	n = 291 134		n = 291 134		n = 5052		n = 20 428		n = 6527		n = 1766		n = 12 252	
Partial F‐statistic of instrument	n/a		30 182.1		103.2		1606.4		302.8		88.7		691.7	

Abbreviations: CI, confidence interval; IV, instrumental variable; SNF, skilled nursing facility.

The instrumental variable regressions, leveraging differences in SNF length of stay related to the copayment change, confirmed the relationship between SNF length of stay and readmission. One more day in a SNF was associated with a 1.5 percentage point lower readmission rate within 30 days of hospital discharge (95% CI −1.6 to −1.4, *P *< .001) and a 1.1 percentage point lower readmission rate within 90 days of hospital discharge (95% CI −1.1 to −1.0, *P *< .001) (Table 4, column 2). This relationship between longer length of SNF stay and better patient outcomes held for readmission within 30 and 90 days of SNF discharge and successful discharge from SNF. In contrast to the ordinary least‐squares results, total Medicare payments for the 90‐day episode after hospital discharge were *higher* in instrumental variable regression, at $396 more with one additional SNF day (95% CI 361‐431, *P *< .001) as were SNF payments (by $591; 95% CI 585‐596, *P *< .001). Total Medicare payment was $179 lower in the 90 days after SNF discharge (95% CI −210 to −149, *P *< .001) These results were consistent across the most common DRGs (Table 4, columns 3‐7).

When stratifying the instrumental variable analyses by comorbidity count, the results remained consistent, suggesting that the main results are not driven by imbalances in patient illness severity (Table S5). They also held when limiting the sample to those with a 30‐ to 60‐day break between SNF stays (Table S6) and when narrowing the range of benefit days on SNF discharge included in the cohort (Table S7).

## 4 DISCUSSION

We found that among Medicare fee‐for‐service beneficiaries, Medicare's SNF copayment policy, with a sharp increase in copayment on the 21st day of a SNF benefit period, was associated with shorter SNF stays. These shorter SNF stays were associated with higher readmission rates, results that were robust across different measures of readmission, study samples, and empirical specifications. We also found that these shorter SNF stays were generally associated with more Medicare spending in the 90‐day period after SNF discharge.

Our results suggest that there are unanticipated and unintended consequences of Medicare's copayment policy in SNF. The large increase in patient copayments on the 21st day of a SNF stay may result in patients being discharged from SNFs sooner than is optimal. This may be because patients cannot bear the financial burden of copayments in excess of $150 per day. It may also be because SNFs do not want to bear the risk of partially uncompensated care after the copayment kicks in. This research suggests that the early discharge associated with copayments results in worse patient outcomes. Our prior research suggests that these effects may be disproportionately born by vulnerable patients—black and Hispanic patients and those with lower socioeconomic status—as vulnerable patients are more likely than their nonvulnerable counterparts to be discharged on their 20th day in a SNF.^6^ This raises concerns that Medicare's copayment policy in SNFs may contribute to disparities in patient outcomes.

We know no prior studies that have directly examined the implications of Medicare's SNF copayment policy or that have estimated the relationship between SNF length of stay and patient outcomes. Our prior descriptive study documented the increased likelihood of discharge on the 20th day of a SNF stay, a phenomenon that was concentrated among vulnerable patients, but this study did not relate this pattern of discharges to patient outcomes or Medicare costs.^15^ Another prior study commented on the relationship between SNF length of stay and patient outcomes by comparing outcomes for Medicare fee‐for‐service beneficiaries with hip fracture to outcomes for similar patients enrolled in Medicare Advantage.^16^ They found that patients enrolled in Medicare Advantage have substantially shorter SNF stays and also lower readmission rates. However, both of these differences could have been driven by the healthier patients enrolled in Medicare Advantage. To our knowledge, our paper provides the first estimates of the relationship between SNF length of stay while accounting for differences in patient characteristics, focusing specifically on the implications of Medicare's copayment policy.

The question of whether keeping patients in a SNF for a longer period of time is beneficial is an important one given the high costs of health care spending, particularly in the postacute care sector, and the fact that SNF payment is per diem, with longer lengths of stay translating into larger payments for any given patient. This extra spending is evidenced in our results, showing that one additional day in a SNF translates into additional spending on the SNF stay of around $600 in most primary specifications. However, this spending may be worthwhile, as it is partially offset by less spending on after discharge from the SNF.

To date, most research has found that cost savings achieved under alternative payment models are driven almost entirely by a decrease in the use of institutional postacute care.^6^, ^7^, ^10^, ^16^ Some studies additionally observed a decline in SNF length of stay under alterative payment systems.^7^, ^8^, ^10^ Indeed, recent research has found that starting in 2015, SNF length of stay started to decline.^4^ Further reductions in postacute care use—both in SNF as a discharge destination and in length of stay once admitted to a SNF—are likely as health systems seek to further tamp down the costs of care and improve the value of the health care they deliver. Prior research has found that discharging patients directly home, instead of to SNF, has adverse consequences for patient outcomes.^7^ These current results suggest that even among patients who are discharged to SNF, declines in SNF length of stay may be accompanied by an increase in rehospitalizations and yield only modest cost savings for insurers.

There are limitations to our research. Our instrumental variable analysis estimates the effect of longer SNF stays only for the marginal patient—those who were discharged by benefit day 20 because of the scheduled copayment increase on day 21. Thus, our results are most relevant in considering the effects of Medicare's SNF cost‐sharing payment rule and suggest it is associated with discharging patients earlier than is otherwise optimal. While our instrumental variable approach improves the internal validity of our results, it also decreases the generalizability of our results. Nonetheless, the results remain consistent across all cohorts we use for estimation and have important implications for Medicare cost‐sharing payment policy. Additionally, we examine the costs associated with longer SNF stays only in monetary terms. Longer SNF stays may have other nonmonetary costs, both positive and negative. Rehospitalizations can be burdensome for patients and families and result in adverse medical consequences such as delirium and hospital‐acquired infections, and longer SNF stays may prevent these outcomes. Longer SNF stays may also result in improved outcomes such as functional status. On the other hand, for patients with a preference for receiving care at home, longer SNF stays delay that preference‐concordant treatment.

Despite these limitations, these findings provide new, robust, and important estimates of the association between Medicare's copayment policy and shorter SNF lengths of stay and, in turn, worse patient outcomes. These findings have important implications for policy and practice, most specifically for the copayment policy we study, but also more generally in the setting of alternative payment models, which have sought in part to constrain health care spending and resulting in reduced institutional postacute care use. Our findings suggest that efforts to reduce health care spending by curtailing postacute care may have adverse consequences for patients. It will be important for future research to identify the types of patients for whom reduced SNF use may result in cost savings without adverse consequences so that the pressure to reduce costs may be better targeted.
