# Supplementary material for: The impact of Medicare copayments for skilled nursing facilities on length of stay, outcomes, and costs
Source: Health Serv Res. 2019 Oct 27;54(6):1184–92. doi: 10.1111/1475-6773.13227 (PMC6863243; doi:10.1111/1475-6773.13227)
Supplement: Supplementary file 2 [file HESR-54-1184-s002.pdf]

Supplementary tables for

“The impact of Medicare copayments for skilled nursing facilities on length of stay, outcomes, and costs”

Rachel M. Werner
R. Tamara Konetzka
Mingyu Qi
Norma B. Coe

In
Health Services Research

Supplemental Table 1. Characteristics of study cohort stratified by instrumental variable (n=291,134)

	**Patients discharged on benefit day 1-6**		**Patients discharged on benefit day 7-14**		**Patients discharged on benefit day 15-40**	
	**n= 94,648**		**n= 100,026**		**n= 96,460**	
Age, mean (SD)	81.3	(8.0)	81.7	(8.1)	82.2	(8.2)
Female, n (%)	54,056	(57.1%)	58,346	(58.3%)	55,966	(58.0%)
Race, n (%)						
-White	85,544	(90.4%)	90,690	(90.7%)	87,200	(90.4%)
-Black	6,825	(7.2%)	7,145	(7.1%)	7,198	(7.5%)
-Hispanic	530	(0.6%)	452	(0.5%)	469	(0.5%)
Married, n (%)	40,034	(42.3%)	40,031	(40.0%)	37,174	(38.5%)
Median household income of ZIP code, mean (SD)	61,342	(24,646)	61,476	(24,610)	61,538	(24,535)
Percent of unemployment of ZIP code, mean (SD)	7.2	(3.5)	7.2	(3.5)	7.3	(3.5)
Percent of population living in poverty of ZIP code, mean (SD)	13.4	(8.2)	13.3	(8.2)	13.3	(8.2)
# of comorbidites, mean (SD)	5.6	(2.8)	5.9	(2.7)	6.3	(2.8)
Total Medicare Part A spending in prior year, mean (SD)	36,022	(33,469)	41,033	(34,769)	48,843	(37,601)
Hospital length of stay, mean (SD)	6.0	(5.1)	6.0	(4.8)	6.2	(5.0)
5 most common diagnosis related groups, n (%)						
-Total knee or hip replacement	1,619	(1.7%)	1,869	(1.9%)	1,564	(1.6%)
-Sepsis	6,155	(6.5%)	6,823	(6.8%)	7,450	(7.7%)
-Urinary tract infection	1,653	(1.8%)	2,230	(2.2%)	2,644	(2.7%)
-Hip fracture	564	(0.6%)	602	(0.6%)	600	(0.6%)
-Congestive heart failure	3,546	(3.8%)	4,170	(4.1%)	4,536	(4.7%)

Abbreviations:
SNF=skilled nursing facility
SD=standard deviation

Supplemental Table 2. Characteristics of high-clinical-severity and low-clinical-severity study cohorts stratified by instrumental variable

	High Clinical Severity Cohort (> 6 comorbidities)						Low Clinical Severity Cohort (≤ 6 comorbidities)					
	Patients discharged on benefit day 1-7		Patients discharged on benefit day 8-15		Patients discharged on benefit day 16-40		Patients discharged on benefit day 1-6		Patients discharged on benefit day 7-14		Patients discharged on benefit day 15-40	
	n=33,382		n= 39,713		n= 43,029		n=61,266		n=60,313		n=53,431	
Age, mean (SD)	79.9	(8.2)	80.4	(8.3)	80.7	(8.4)	82.1	(7.8)	82.5	(7.9)	83.3	(7.9)
Female, n (%)	20,324	(52.1%)	20,542	(53.7%)	20,777	(53.5%)	36,745	(60.0%)	36,996	(61.3%)	32,984	(61.7%)
Race, n (%)												
-White	34,663	(88.8%)	34,093	(89.1%)	34,520	(88.9%)	55,901	(91.2%)	55,337	(91.8%)	48,920	(91.6%)
-Black	3,368	(8.6%)	3,268	(8.5%)	3,453	(8.9%)	3,956	(6.5%)	3,707	(6.2%)	3,416	(6.4%)
-Hispanic	218	(0.6%)	181	(0.5%)	190	(0.5%)	334	(0.6%)	278	(0.5%)	250	(0.5%)
Married, n (%)	17,441	(44.7%)	16,206	(42.4%)	15,998	(41.2%)	25,044	(40.9%)	23,096	(38.3%)	19,454	(36.4%)
Median household income of ZIP code, mean (SD)	60,083	(24,007)	60,526	(24,127)	60,770	(24,122)	62,028	(24,961)	62,101	(24,902)	62,157	(24,846)
Percent of unemployment of ZIP code, mean (SD)	7.4	(3.6)	7.4	(3.6)	7.4	(3.6)	7.1	(3.4)	7.1	(3.4)	7.1	(3.4)
Percent of population living in poverty of ZIP code, mean (SD)	13.8	(8.4)	13.7	(8.4)	13.6	(8.4)	13.2	(8.1)	13.1	(8.0)	13.1	(8.1)
# of comorbidites, mean (SD)	8.6	(1.6)	8.7	(1.7)	8.8	(1.8)	3.9	(1.7)	4.1	(1.6)	4.2	(1.5)
Total Medicare Part A spending in prior year, mean (SD)	52,160	(42,519)	55,459	(42,165)	63,458	(45,203)	27,230	(22,940)	31,534	(24,635)	37,073	(24,421)
Hospital length of stay, mean (SD)	6.7	(5.8)	6.6	(5.3)	6.8	(5.4)	5.7	(4.7)	5.6	(4.5)	5.8	(4.6)
5 most common diagnosis related groupss, n (%)												
-Total knee or hip replacement	111	(0.3%)	155	(0.4%)	183	(0.5%)	1,527	(2.5%)	1,714	(2.8%)	1,362	(2.6%)
-Sepsis	3,253	(8.3%)	3,167	(8.3%)	3,567	(9.2%)	3,365	(5.5%)	3,543	(5.9%)	3,533	(6.6%)

-Urinary tract infection	521	(1.3%)	657	(1.7%)	765	(2.0%)	1,224	(2.0%)	1,556	(2.6%)	1,804	(3.4%)
-Hip fracture	111	(0.3%)	146	(0.4%)	134	(0.4%)	468	(0.8%)	454	(0.8%)	453	(0.9%)
-Congestive heart failure	2,400	(6.2%)	2,452	(6.4%)	2,650	(6.8%)	1,491	(2.4%)	1,648	(2.7%)	1,611	(3.0%)

Abbreviations:
SNF=skilled nursing facility
SD=standard deviation

Supplemental Table 3. Characteristics of study cohort stratified by instrumental variable, limiting the study cohort to those with 30 to 60 days between current SNF and prior SNF stay

	Patients discharged on benefit day 1-11		Patients discharged on benefit day 12-18		Patients discharged on benefit day 19-40	
	n=10,145		n=10,006		n=10,172	
Age, mean (SD)	80.9	(8.0)	81.8	(8.1)	82.3	(8.0)
Female, n (%)	5,968	(58.8%)	6,215	(62.1%)	6,245	(61.4%)
Race, n (%)						
-White	9,358	(92.2%)	9,289	(92.8%)	9,300	(91.4%)
-Black	572	(5.6%)	537	(5.4%)	666	(6.6%)
-Hispanic	48	(0.5%)	40	(0.4%)	50	(0.5%)
Married, n (%)	4,170	(41.1%)	3,703	(37.0%)	3,722	(36.6%)
Median household income of ZIP code, mean (SD)	63,011	(25,285)	63,697	(25,528)	62,603	(25,089)
Percent of unemployment of ZIP code, mean(SD)	7.1	(3.3)	7.0	(3.3)	7.1	(3.4)
Percent of population living in poverty of ZIP code, mean(SD)	12.9	(8.0)	12.7	(7.9)	13.0	(8.2)
# of comorbidites, mean (SD)	5.8	(2.9)	5.6	(2.8)	5.9	(2.8)
Total Medicare Part A spending in prior year, mean (SD)	42,742	(40,304)	41,453	(31,820)	47,561	(33,659)
Hospital length of stay, mean (SD)	8.7	(9.0)	7.0	(6.4)	7.3	(6.4)
5 most common diagnosis related groups, n (%)						
-Total knee or hip replacement	692	(6.8%)	749	(7.5%)	404	(4.0%)
-Sepsis	599	(5.9%)	563	(5.6%)	662	(6.5%)
-Urinary tract infection	240	(2.4%)	290	(2.9%)	353	(3.5%)
-Hip fracture	134	(1.3%)	101	(1.0%)	118	(1.2%)
-Congestive heart failure	327	(3.2%)	343	(3.4%)	383	(3.8%)

Abbreviations:
SNF=skilled nursing facility
SD=standard deviation

Supplemental Table 4. Characteristics of study cohort stratified by instrumental variable, limiting the study cohort to those patients discharged on benefit day 16-25

	**Patients discharged on benefit day 1-6**		**Patients discharged on benefit day 7-12**		**Patients discharged on benefit day 13-25**	
	**n=31,240**		**n=32,574**		**n=27,728**	
Age, mean (SD)	81.1	(8.0)	81.3	(8.1)	81.6	(8.3)
Female, n (%)	18,180	(58.2%)	18,809	(57.7%)	15,437	(55.7%)
Race, n (%)						
-White	28,121	(90.0%)	29,498	(90.6%)	24,916	(89.9%)
-Black	2,340	(7.5%)	2,364	(7.3%)	2,218	(8.0%)
-Hispanic	190	(0.6%)	175	(0.5%)	134	(0.5%)
Married, n (%)	13,140	(42.1 %)	13,383	(41.1%)	11,082	(40.0%)
Median household income of ZIP code, mean (SD)	61,241	(24,612)	61,255	(24,639)	60,256	(24,191)
Percent of unemployment of ZIP code, mean (SD)	7.2	(3.5)	7.3	(3.5)	7.3	(3.6)
Percent of population living in poverty of ZIP code, mean (SD)	13.5	(8.3)	13.4	(8.2)	13.7	(8.3)
# of comorbidites, mean (SD)	5.5	(2.8)	5.9	(2.8)	6.3	(2.8)
Total Medicare Part A spending in prior year, mean (SD)	34,798	(31,665)	39,891	(33,369)	46,485	(37,209)
Hospital length of stay, mean (SD)	5.9	(4.8)	5.9	(4.6)	6.4	(5.3)
5 most common Diagnosis related groups, n (%)						
-Total knee or hip replacement	541	(1.7%)	722	(2.2%)	374	(1.4%)
-Sepsis	1,851	(5.9%)	2,138	(6.6%)	2,259	(8.2%)
-Urinary tract infection	582	(1.9%)	703	(2.2%)	618	(2.2%)
-Hip fracture	186	(0.6%)	170	(0.5%)	154	(0.6%)
-Congestive heart failure	1,133	(3.6%)	1,345	(4.1%)	1,302	(4.7%)

Abbreviations:
SNF=skilled nursing facility
SD=standard deviation

Supplemental Table 5. Differences in outcomes with one additional day in SNF from instrumental variable regressions, stratified by clinical severity

	**High Clinical Severity Cohort (> 6 comorbidities)**		**Low Clinical Severity Cohort (≤ 6 comorbidities)**	
	**Difference**	**P-value**	**Difference**	**P-value**
	**(95% CI)**		**(95% CI)**	
Readmission within 30 days of hospital discharge, percentage points	-1.6	<.001	-1.3	<.001
	(-1.7 to -1.5)		(-1.4 to -1.2)	
	n=116,124		n=175,010	
Readmission within 90 days of hospital discharge, percentage points	-1.0	<.001	-1.0	<.001
	(-1.1 to -0.9)		(-1.1 to -0.9)	
	n=116,124		n=175,010	
Readmission within 30 days of SNF discharge, percentage points	-0.9	<.001	-0.9	<.001
	(-1.0 to -0.7)		(-1.0 to -0.8)	
	n=102,079		n=161,317	
Readmission within 90 days of SNF discharge, percentage points	-1.0	<.001	-1.0	<.001
	(-1.2 to -0.9)		(-1.1 to -0.9)	
	n=102,079		n=161,317	
Successful discharge from SNF	1.3	<.001	1.4	<.001
	(1.2 to 1.4)		(1.3 to 1.5)	
	n=102,079		n=161,317	
Total Medicare payment in first 90 days after hospital discharge, $	387	<.001	512	0.021
	(329 to 445)		(453 to 572)	
	n=116,124		n=175,010	
Medicare payment for index hospitalization, $	88	<.001	155	<.001
	(70 to 106)		(110 to 200)	

	n=116,124		n=175,010	
Medicare payment for index SNF stay, $	588	<.001	603	<.001
	(579 to 596)		(595 to 611)	
	n=116,124		n=175,010	
Total Medicare payment within first 90 days after SNF discharge, $	-136	<.001	-156	<.001
	(-194 to -79)		(-194 to -118)	
	n=116,124		n=175,010	
Partial F-statistic of instrument	11,631.6		19,398.9	

Abbreviations:
CI=confidence interval
SNF=skilled nursing facility

Supplemental Table 6. Differences in outcomes with one additional day in SNF from instrumental variable regressions, using study cohort limited to those with 30 to 60 days between current SNF and prior SNF stay

	Difference	P-value
	(95% CI)	
Readmission within 30 days of hospital discharge, percentage points	-2.2	<.001
	(-2.5 to -1.9)	
	n=30,323	
Readmission within 90 days of hospital discharge, percentage points	-1.2	<.001
	(-1.6 to -0.9)	
	n=30,323	
Readmission within 30 days of SNF discharge, percentage points	-1.5	<.001
	(-1.9 to -1.1)	
	n=28,523	
Readmission within 90 days of SNF discharge, percentage points	-1.3	<.001
	(-1.7 to -0.9)	
	n=28,523	
Successful discharge from SNF	2.3	<.001
	(1.9 to 2.6)	
	n=28,523	
Total Medicare payment in first 90 days after hospital discharge, $	417	<.001
	(277 to 558)	
	n=30,323	
Medicare payment for index hospitalization, $	244	<.001
	(173 to 314)	
	n=30,323	
Medicare payment for index SNF stay, $	614	<.001
	(593 to 635)	
	n=30,323	
Total Medicare payment within first 90 days after SNF discharge, $	-288	<.001
	(-416 to -160)	
	n=30,323	
Partial F-statistic of instrument	1,621.7	

Abbreviations:
CI=confidence interval
SNF=skilled nursing facility

Supplemental Table 7. Differences in outcome with one additional day in SNF from instrumental variable regressions, using study cohort limited to those discharged on benefit day 16-25

	Difference	P-value
	**(95% CI)**	
Readmission within 30 days of hospital discharge, percentage points	-2.0	<.001
	(-2.1 to -1.9)	
	n=91,542	
Readmission within 90 days of hospital discharge, percentage points	-1.5	<.001
	(-1.6 to -1.4)	
	n=91,542	
Readmission within 30 days of SNF discharge, percentage points	-1.5	<.001
	(-1.6 to -1.4)	
	n=82,965	
Readmission within 90 days of SNF discharge, percentage points	-1.5	<.001
	(-1.5 to -1.4)	
	n=82,965	
Successful discharge from SNF	1.9	<.001
	(1.8 to 1.9)	
	n=82,965	
Total Medicare payment in first 90 days after hospital discharge, $	-8	<.001
	(-45 to 30)	
	n=91,542	
Medicare payment for index hospitalization, $	61	<.001
	(40 to 81)	
	n=91,542	
Medicare payment for index SNF stay, $	479	<.001
	(474 to 483)	
	n=91,542	
Total Medicare payment within first 90 days after SNF discharge, $	-444	<.001
	(-476 to -412)	
	n=91,542	
Partial F-statistic of instrument	172,540.5	

Abbreviations:
CI=confidence interval
SNF=skilled nursing facility
